# Supplementary material for: Bacillus cereus biovar anthracis causes inhalational anthrax-like disease in rabbits that is treatable with medical countermeasures
Source: PLoS Negl Trop Dis. 2025 Apr 7;19(4):e0012973. doi: 10.1371/journal.pntd.0012973 (PMC12005533; doi:10.1371/journal.pntd.0012973)
Supplement: S1 Data — (PDF) [file pntd.0012973.s001.pdf]

Raw data, *Bacillus cereus* biovar *anthracis* causes inhalational anthrax-like disease in rabbits that is treatable with medical countermeasures

Fig 2 Dose-response in rabbits exposed by inhalation to Bcbva CA and CI spores. Particle size distributions of purified spores during aerosol exposures were measured by the APS. The mass median aerodynamic diameter (MMAD) for each set of animal exposures is indicated by individual dots (**A**). The horizontal line represents the replicate mean and corresponding geometric standard deviation. Probit dose-response (solid line) with 85% confidence intervals (dashed lines) are shown for Bcbva CA (**B**) and CI (**C**).

Fig 2A MMAD Bcbva CA

MMAD (ALL)	MMAD (Test 1)	MMAD (Test 2)	MMAD (Test 3)	MMAD (Test 4)	MMAD (Test 5)
1.336	1.336	1.354	1.266	1.577*	1.304
1.32	1.32	1.328	1.256	1.265	1.296
1.324	1.324	1.337	1.245	1.267	1.288
1.361	1.361	1.333	1.242	1.248	1.281
1.354					
1.328					
1.337					
1.333					
1.266					
1.256					
1.245					
1.242					
1.577*					
1.265					
1.267					
1.248					
1.304					
1.296					
1.288					
1.281					

Fig 2A MMAD Bcbva CI

MMAD (ALL)	MMAD (Test 6)	MMAD (Test 7)	MMAD (Test 8)	MMAD (Test 9)	MMAD (Test 10)
1.403	1.403	1.37	1.416	1.332	1.378
1.4	1.4	1.376	1.424	1.326	1.369
1.402	1.402	1.376	1.419	1.327	1.358
1.402	1.402	1.363	1.406	1.315	1.352
1.37					
1.376					
1.376					

1.363
1.416
1.424
1.419
1.406
1.332
1.326
1.327
1.315
1.378
1.369
1.358
1.352

Fig 2B Dose response Bcbva CA

Inhaled dose	Number challenged	Number succumb
2.70E+04	8	1
7.90E+04	8	1
3.20E+05	8	5
4.80E+06	8	8
3.90E+07	8	8

Fig 2C Dose response Bcbva CI

Inhaled dose	Number challenged	Number succumb
1.80E+05	16	4
6.30E+05	8	6
1.90E+06	8	7
4.90E+07	8	8

Fig. 3. Representative temperature responses in rabbits exposed by inhalation to Bcbva CI spores. Raw data uploaded as a separate file.

Fig 4. SIBT and time to death in rabbits exposed by inhalation to Bcbva CA and CI spores. The time to SIBT following aerosol exposure was recorded for each animal and plotted as the percentage of animals with normal body temperature per dosage group (**A**, **B**). The time of death for animals that succumbed to infection was plotted as percent survival per dosage group (**C**, **D**). Two groups of 8 animals were challenged with 1.8 X 10^5 CFU Bcbva CI. These groups were combined for data analysis (16 animals in this group).

Fig 4A Time to SIBT Bcbva CA

	Dose				
Time(h)	3.2 x 10^5CFU	4.8 x 10^6CFU	2.7 x 10^4CFU	7.9 x 10^4CFU	3.9 x 10^7CFU
504	0				
504	0				
44.25	1				
504	0				
241.5	1				
56	1				
288.25	1				
45	1				
354.75		1			
59.75		1			
37.5		1			
49		1			
34.5		1			
236.25		1			
149.5		1			
504			0		
504			0		
504			0		
504			0		
504			0		
504			0		
417.5			1		
504			0		
504				0	
504				0	
250				1	
504				0	
504				0	
504				0	
504				0	

504				0	
35.5					1
46.25					1
40.25					1
43.25					1
38.5					1
32.5					1
52.75					1
33.75					1

Fig 4B Time to SIBT Bcbva CI

			Dose	
Time(h)	6.3 x 10^5CFU	1.8 x 10^5CFU	1.9 x 10^6CFU	4.9 x 10^7 CFU
68.25	1			
86.5	1			
103	1			
92.25	1			
504	0			
504	0			
42	1			
64.5	1			
504		0		
504		0		
504		0		
504		0		
504		0		
504		0		
504		0		
504		0		
63.5			1	
51.25			1	
164			1	
504			0	
45.25			1	
42.75			1	
37			1	
504				0
504				0
504				0
57.25				1

149.25	1	
504	0	
224.25	1	
38.25		1
47.5		1
40		1
38.75		1
28.75		1
40.25		1
48.5		1
44.5		1

Fig 4C Time to Death Bcbva CA

	Dose				
Time(h)	3.2×10^5CFU	4.8×10^6CFU	2.7×10^4CFU	7.9×10^4CFU	3.9×10^7CFU
504	0				
504	0				
72.25	1				
504	0				
300.75	1				
106	1				
294	1				
84.25	1				
374.5		1			
66.25		1			
62.25		1			
86.25		1			
64.5		1			
248.5		1			
181		1			
504			0		
504			0		
504			0		
504			0		
504			0		
504			0		
436.75			1		
504			0		
504				0	
504				0	
295.25				1	

504				0	
504				0	
504				0	
504				0	
504				0	
61.75					1
58					1
83					1
49.5					1
50.75					1
42.75					1
64					1
53.5					1

Fig 4D Time to Death Bcbva CI

	Dose			
Time(h)	6.3 x 10^5CFU	1.8 x 10^5CFU	1.9 x 10^6CFU	4.9 x 10^7 CFU
71.25	1			
105.75	1			
160.75	1			
106.75	1			
504	0			
504	0			
56.25	1			
76	1			
504		0		
504		0		
504		0		
504		0		
504		0		
504		0		
504		0		
504		0		
70.5			1	
61.5			1	
200.25			1	
504			0	
49.75			1	
51.25			1	
82.5			1	

504	0	
504	0	
504	0	
69.75	1	
153	1	
504	0	
252	1	
43.5		1
121		1
48.27		1
53		1
57.5		1
49		1
65.5		1
60.75		1

Table 1. Dose-dependent median time to death following inhalation exposure to Bcbva CA and CI. Log-Rank Mantel Cox Test was performed to identify differences in time to death among the administered doses. Data were derived from 8 animals per dose of Bcbva CA and CI and 10 animals for Ames A2084. Since multiple deaths/dose group are required to determine a median time to death, only the data for those doses are listed. The *B. anthracis* data were derived from a previous study (25). Since dose does not influence time to death for *B. anthracis* challenge, the dose range and mean time to death are reported. **Raw data are shown in Fig 4 above**.

Fig 5. Longitudinal evaluation of bacteremia following Bcbva inhalation spore challenge (animal died on day 0). Symbols indicate each data point, and time points are relative to the time of death in days (d). Mean titers (horizontal line) are shown. The sampling results are shown for animals with at least two consecutive days of sampling (n = 16 for CA; n = 21 for CI). Only quantifiable titers are shown. The absence of a data point indicates no detectable bacterial growth observed from the sample of corresponding animals.

day	Bcbva CA (CFU/mL)																				
-2			267			66.7						500				133					
-1	200	133	66.7	200		667	16700	733	433	1070	367	633			9300						
0	2.00E+07	1.97E+07	8.70E+05	3.37E+07	1.73E+07	3.40E+06	1.52E+07	3.00E+06	5.73E+05	1.70E+07	1.09E+08	1.35E+07	7.57E+07	7.10E+07	3.53E+07	1.08E+08					
	Bcbva CI (CFU/mL)																				
-2											33.3										
-1		3200	66.7	6870				90700	133		1730		2030		133						
0	4.17E+08	8.57E+06	1.06E+08	7.60E+07	5.30E+07	4.27E+08	3.43E+07	8.07E+07	1.47E+07	2.63E+08	1.01E+06	1.02E+08	8.27E+05	3.67E+08	2.70E+06	1.28E+07	1.37E+07	1.85E+08	5.43E+07	3.33E+07	1.97E+07

Table 2. Terminal bacterial burden in animals infected with Bcbva. Animals that succumbed to infection in the LD_{50} studies (n = 23 for CA; n = 25 for CI) were sampled, and mean CFU/ml or g determined for blood, BAL, liver, lung, and spleen tissues. Six survivors were sampled as controls and bacteria were not observed in any of the assayed tissues or fluids at 28 days post-exposure. Data shown are mean with lower and upper 95% confidence intervals in brackets.

Bcbva CA				
Blood (CFU/mL)	BAL (CFU/mL)	Lung (CFU/g)	Liver (CFU/g)	Spleen (CFU/g)
3.37E+07	1.29E+05	3.34E+07	1.66E+08	3.29E+07
1.73E+07	1.03E+05	5.76E+07	8.22E+06	2.98E+07
3.40E+06	8.73E+05	2.41E+08	2.74E+07	4.79E+07
1.52E+07	4.97E+04	1.04E+08	1.22E+08	5.80E+07
3.00E+06	1.33E+05	1.10E+08	6.06E+07	7.10E+07
5.73E+05	1.83E+04	5.94E+07	1.16E+07	3.56E+07
4.20E+07	3.73E+05	8.72E+07	4.88E+07	7.80E+07
8.77E+06	6.93E+05	3.00E+07	6.71E+07	7.00E+07
2.00E+07	2.83E+04	5.76E+08	1.45E+08	9.57E+07
8.70E+05	7.17E+03	6.86E+07	5.14E+07	4.57E+08
1.97E+07	4.30E+03	4.00E+07	7.67E+07	1.19E+08
4.20E+07	9.57E+04	1.54E+06	7.70E+07	1.04E+08
2.87E+07	1.24E+05	3.79E+07	1.28E+07	3.13E+06
5.63E+07	1.57E+03	2.05E+08	2.74E+08	8.00E+07
7.57E+07	7.93E+04	1.50E+08	2.77E+08	4.91E+07
7.10E+07	1.97E+03	1.60E+08	3.80E+08	5.81E+07
1.17E+07	9.17E+04	1.15E+08	4.39E+08	5.78E+07
1.09E+08	2.37E+06	7.86E+08	2.78E+08	1.06E+08
3.53E+07	1.77E+04	6.83E+07	5.67E+07	6.29E+07
1.08E+08	3.90E+04	3.35E+07	4.89E+07	5.17E+07
1.35E+07	4.20E+04	4.51E+07	6.93E+07	1.68E+07
8.13E+06	1.55E+04	2.08E+07	5.08E+06	2.38E+07
1.26E+06	9.97E+03	2.21E+07	4.63E+07	1.12E+08

Bcbva CI				
Blood (CFU/mL)	BAL (CFU/mL)	Lung (CFU/g)	Liver (CFU/g)	Spleen (CFU/g)
4.17E+08	5.13E+04	5.33E+08	4.07E+08	1.17E+08
8.57E+06	4.77E+04	2.47E+07	3.48E+07	6.61E+07
7.60E+07	1.16E+06	1.05E+09	7.68E+08	7.73E+08
1.06E+08	3.07E+04	9.75E+07	4.29E+07	1.85E+08
5.07E+07	9.53E+04	2.45E+07	1.07E+08	5.29E+08
1.26E+07	2.70E+04	1.28E+08	4.81E+07	8.62E+07
8.07E+07	5.13E+05	6.40E+07	4.00E+08	1.11E+08
2.63E+08	3.50E+05	1.29E+09	3.19E+08	3.50E+08
4.27E+08	7.37E+04	8.33E+08	4.28E+08	1.24E+07
3.43E+07	4.83E+05	1.62E+08	6.87E+07	3.19E+07
2.27E+07	7.70E+03	1.18E+08	8.83E+07	8.43E+07
1.01E+08	5.83E+04	1.03E+08	5.38E+07	8.93E+07
4.77E+07	1.60E+04	2.46E+07	4.67E+07	6.30E+07
1.02E+08	7.03E+05	1.30E+08	1.70E+08	9.33E+07
4.70E+07	6.57E+04	3.22E+07	3.33E+04	7.33E+07
8.27E+07	3.77E+05	6.80E+06	4.67E+04	5.95E+07
3.53E+07	7.00E+04	1.98E+07	6.33E+07	2.71E+07
3.67E+08	1.10E+05	1.45E+08	3.18E+08	5.00E+07
1.28E+07	9.37E+05	4.78E+08	2.35E+08	6.67E+08
5.43E+07	4.20E+04	1.73E+08	1.38E+08	6.15E+08
1.37E+07	1.22E+04	7.47E+07	5.68E+07	1.51E+07
1.85E+08	1.38E+04	2.80E+08	1.26E+08	2.50E+07
1.97E+07	4.97E+04	6.33E+07	8.87E+07	7.19E+07
3.33E+07	8.07E+04	2.75E+07	8.43E+07	3.63E+07
2.70E+06	4.93E+04	5.61E+08	3.87E+08	3.97E+08

Fig 6. Longitudinal evaluation of Bcbva CA virulence factor expression (animal died on day 0). Hyaluronic acid (**A**) and protective antigen (**B**) concentrations were measured in serum samples each day for five days following exposure to Bcbva CA spores. Symbols indicate individual data points. Horizontal line indicates the mean. Time points are relative to the time of death (d) for animals that succumbed to aerosol exposure within the first five days of the observation period. The dashed horizontal lines indicate the mean virulence factor expression levels in all samples from survivors for the first 5 days following aerosol exposure, since no statistical increase was observed for survivors during this period. For non-survivors, statistical significance of the level of virulence factor expression was determined using ANOVA analysis followed by a Tukey's multiple comparisons test as described in the methods. * indicates a P-value of< 0.05.

Fig 6A

day	non-survivors hyaluronic acid (ng/mL)					
-4			41.49	15.71		25.80
-3	32.99	33.83	30.06	24.46	40.84	9.37
-2	8.73	24.21	37.00	20.03	26.06	37.26
-1	21.28	12.20	2097.72	59.05	62.03	130.17
0	1.59E+06	5.10E+05	4.54E+05	1.72E+06	1.44E+06	6.01E+05

Fig 6B

day	non-survivors protective antigen (ng/mL)					
-4			35.86	5.89		19.97
-3	19.26	18.02	32.82	0.3	12.62	16.31
-2	20.63	16.14	41.36	9.51	6.82	15.59
-1	24.93	13.25	144.32	17.3	6.61	51.43
0	1.05E+04	3.16E+03	3.90E+03	1.69E+04	5.42E+03	1.51E+04

Fig 7. Longitudinal evaluation of Bcbva CI virulence factor expression (animal died on day 0). Hyaluronic acid (**A**) and protective antigen (**B**) concentrations were measured in serum samples each day for five days following exposure to Bcbva CI spores. Symbol indicates data point. Horizontal line indicates the mean. Time points are relative to the time of death (d) for the analyzed animals that succumbed to aerosol exposure within the first five days of the observation period. The dashed horizontal lines indicate the mean virulence factor expression levels in all samples from survivors for the first 5 days following exposure since no statistical increase was observed for survivors during this period. For non-survivors, statistical significance of the level of virulence factor expression was determined using ANOVA analysis followed by a Tukey's multiple comparisons test as described in the methods. * indicates a P-value of < 0.05.

Fig 7A

day	non-survivors hyaluronic acid (ng/mL)							
-4								
-3			27.54	23.37	26.80	26.46	34.80	7.72
-2	8.81	21.68	20.52	28.12	40.32	3.58	17.14	7.24
-1	4.97	9.43	21.36	49.16	25.09	7.94	24.40	14.77
0	9.89E+05	4.52E+05	1.40E+05	6.52E+05	5.78E+05	1.18E+06	9.68E+05	4.25E+05

Fig 7B

day	non-survivors protective antigen (ng/mL)							
-4								
-3			9.64	19.49	14.56	7.13	16.76	29.81
-2	25.20	0.10	15.15	19.02	12.67	3.22	13.22	23.67
-1	21.97	0.10	12.06	20.00	18.69	9.24	1.40	32.47
0	1.94E+04	1.77E+04	3.89E+03	7.22E+03	1.88E+04	1.03E+04	1.20E+04	3.92E+03

Fig 8. *In vitro* versus terminal *in vivo* virulence factor expression. Data represents the mean and standard deviation of the analyzed biological replicates (three replicates for *in vitro*; 10 Bcbva CA and 14 Bcbva CI replicates each for *in vivo*) for hyaluronic acid (**A**), protective antigen (**B**), and PDGA capsule (**C**) at stationary growth (*in vitro*) or in terminal blood samples (*in vivo*), which would constitute maximal growth in the respective conditions. The amount of HA in the *B. anthracis* samples was not statistically different from a negative control. HA was not detected in serum collected from animals prior to challenge with Bcbva CA or CI.

Fig 8A

hyaluronic acid (ng/mL) stationary phase in vitro or terminal in vivo				
In vitro CA	In vitro CI	In vitro B. anthracis	In vivo CA	In vivo CI
3421.35	3169.00	3.09	1.59E+06	9.89E+05

3421.35	2916.76	0.00	5.10E+05	4.52E+05
3376.49	3112.06	9.69	4.54E+05	1.40E+05
			1.72E+06	6.52E+05
			1.44E+06	5.78E+05
			6.01E+05	1.18E+06
			1.34E+06	9.68E+05
			5.00E+05	4.25E+05
			5.71E+05	1.66E+06
			1.45E+05	1.37E+06
				2.05E+05
				4.56E+05
				1.29E+06

Fig 8B

protective antigen (ng/mL) stationary phase in vitro or terminal in vivo				
In vitro CA	In vitro CI	In vitro B. anthracis	In vivo CA	In vivo CI
3405.55	352.28	3973.47	1.05E+04	1.94E+04
4874.31	307.38	2714.07	3.16E+03	1.77E+04
		4254.10	3.90E+03	3.89E+03
			1.69E+04	7.22E+03
			5.42E+03	1.88E+04
			1.51E+04	1.03E+04
			1.52E+03	1.20E+04
			4.45E+03	3.92E+03
			1.65E+03	3.21E+02
			2.93E+03	2.25E+04
				2.03E+04
				1.26E+03
				1.07E+04

Fig 8C

capsule (ng/mL) stationary phase in vitro or terminal in vivo				
In vitro CA	In vitro CI	In vitro B. anthracis	In vivo CA	In vivo CI
3.77E+05	4.01E+05	1.69E+05	5.69E+05	1.12E+06

4.08E+05	4.32E+05	1.93E+05	2.37E+06	1.12E+06
4.28E+05	4.48E+05	1.92E+05	9.41E+05	7.89E+05
			2.27E+06	1.93E+05
			1.14E+06	1.33E+06
			1.36E+06	8.28E+05
			1.38E+06	7.99E+05
			1.62E+06	1.48E+06
				1.67E+06
				1.87E+06
				2.46E+05
				4.90E+05
				1.81E+06

Fig 9. Temperature responses and time to death as a function of treatment following Bcbva CA aerosol exposure. Time to SIBT (A), time to death (B), magnitude of SIBT (peak and mean; C and D, respectively), number of SIBT (E), and cumulative duration of SIBT (F)] are indicated. Post indicates post-exposure prophylaxis (PEP). The time to SIBT for post levofloxacin-treated animals was determined from cessation of treatment. If the animal did not display SIBT or survived the 28-day observation period, 672.00 h was used as the time to SIBT and death, respectively. + Indicates treatment occurred. – Indicates treatment did not occur. One-way ANOVA was performed followed by a Dunnett's Test to perform pair-wise comparisons between the MCM treatment groups and control. * Indicates $P < 0.05$. ** Indicates $P < 0.01$. *** Indicates $P < 0.001$. **** Indicates $P < 0.0001$. Only statistically significant comparisons are shown.

	Fig 9A					Fig 9B				Fig 9C			
		Time to SIBT (h)				Time to Death (h)				Peak SIBT Temp (degrees above baseline)			
		35.50	68.50	61.75	131.00	61.75	166.72	672.00	672.00	2.01	2.69	2.65	2.36
		38.50	155.25	122.00	143.00	58.00	116.00	672.00	672.00	1.79	2.82	2.01	2.33
		46.25	87.50	65.25	35.75	83.00	142.75	672.00	672.00	2.43	2.78	2.92	2.91
		32.50	77.75	88.75	44.00	49.50	672.00	672.00	672.00	2.47	2.85	2.79	2.82
		40.25	85.50	155.00	47.25	53.75	116.00	672.00	672.00	2.79	2.84	1.94	3.01
		52.75	84.50	86.25	70.50	54.75	131.25	672.00	672.00	2.67	2.83	2.6	2.19
		43.25	*392.75**	113.50	37.00	69.00	119.00	672.00	672.00	2.46	3.06	1.97	2.32
		33.75	72.25	86.25	57.75	77.25	73.00	672.00	672.00	2.29	2.82	2.77	3.36
		35.75	83.50	101.00	70.75	50.75	232.75	672.00	672.00	2.53	2.46	2.37	2.89
		53.50	73.75	114.25	44.75	49.25	115.00	672.00	672.00	2.19	2.68	2.61	3.58
		41.00	97.00	185.00	59.00	50.75	225.75	672.00	672.00	2.83	2.88	2.24	3.7
		44.50	86.50	150.25	35.75	42.75	159.75	672.00	672.00	2.51	3.09	2.79	2.78
		43.75	77.25	122.25	99.00	64.00	126.25	672.00	672.00	3.14	3.08	2.08	3.51
		38.50	119.50		32.00	53.50	159.50	672.00	672.00	2.31	2.73		2.86
		39.75	64.75		73.00	73.00	120.50	672.00	672.00	3.57	2.7		2.97
		37.75	100.00		45.50	59.75	124.25	672.00	672.00	2.02	2.58		2.57
		40.25	167.50		64.75	47.00	197.00	672.00	672.00	2.57	2.49		3.54
		30.50	124.75		40.25	50.50	158.50	672.00	672.00	2.41	3.58		3.06
		32.25	60.75		85.00	76.50	204.00		672.00	3.28	3.01		2.71
		38.50	93.00			61.50	135.75		672.00	1.83	2.34		
Post-levofloxacin		−	+	+	−	−	+	+	−	−	+	+	−
Post-AVA		−	−	+	−	−	−	+	−	−	−	+	−
Pre-AVA		−	−	−	+	−	−	−	+	−	−	−	+

	Fig 9D					Fig 9E				Fig 9D			
		Mean SIBT (degrees above baseline)				Number of SIBTs				SIBT duration			
		1.63	1.65	1.78	1.59	2	1	3	2	6.00	66.00	21.75	11.50
		1.57	1.81	1.36	1.36	1	1	2	5	14.00	60.75	19.75	30.75
		1.88	1.99	1.80	1.82	1	2	2	3	11.25	40.00	25.50	57.75
		2.02	1.97	1.62	1.64	1	2	4	6	8.00	59.50	37.00	65.00
		1.96	1.90	1.46	1.80	1	1	2	2	20.50	38.50	20.00	48.25
		1.99	2.11	1.72	1.46	1	1	1	2	5.75	40.25	26.75	17.75
		1.88	1.88	1.43	1.84	1	2	1	5	21.00	60.75	21.25	27.75
		1.97	1.62	1.93	2.25	1	3	3	4	8.00	54.75	11.50	70.50
		1.87	1.66	1.91	1.58	1	1	2	5	5.50	27.25	24.25	79.25
		1.74	1.83	1.70	2.22	3	1	1	4	*38.25**	37.50	23.50	79.50
		2.31	2.18	1.58	2.50	1	1	1	4	5.25	30.25	12.50	50.75
		1.88	2.34	2.07	2.33	1	1	2	2	19.50	34.00	14.00	22.00
		2.34	2.37	1.49	2.38	2	1	2	2	15.50	34.50	35.75	27.75
		1.47	1.99		1.76	2	3		5	7.75	48.00		50.00
		2.84	2.13		2.02	1	1		2	10.50	6.75		35.25
		1.36	1.54		1.63	1	2		2	8.00	38.50		44.50
		1.97	1.68		2.11	1	2		5	20.50	48.25		44.00
		1.91	2.10		2.12	1	3		5	8.50	48.75		61.00
		2.87	2.41		1.98	1	2		2	4.25	35.00		29.25
		1.58	1.39			1	2			4.50	25.50		
Post-levofloxacin		−	+	+	−	−	+	+	−	−	+	+	−
Post-AVA		−	−	+	−	−	−	+	−	−	−	+	−
Pre-AVA		−	−	−	+	−	−	−	+	−	−	−	+

Fig 10. Temperature responses and time to death as a function of treatment following Bcbva CI aerosol exposure. Temperature response [time to SIBT (A), time to death (B), magnitude of SIBT (peak and mean; C and D, respectively), number of SIBT (E), and cumulative duration of SIBT (F)] are indicated. Post indicates post-exposure prophylaxis (PEP). The time to SIBT for levofloxacin treated animals was determined from cessation of treatment. If the animal did not display SIBT or survived the 28-day observation period, 672.00 used as the time to SIBT and death, respectively. + Indicates treatment occurred. – indicates treatment did not occur. One-way ANOVA was performed followed by a Dunnett's Test to perform pair-wise comparisons between the MCM treatment groups and control. * Indicates P < 0.05. ** Indicates P < 0.01. *** Indicates P < 0.001. **** Indicates P < 0.0001. Only statistically significant comparisons are shown.

	Fig 10A				Fig 10B				Fig 10C			
	Time to SIBT (h)				Time to Death (h)				Peak SIBT Temp (degrees above baseline)			
	38.25	48.00	193.00	36.50	43.50	190.25	672.00	672.00	2.48	2.74	1.71	2.85
	28.75	65.00	101.75	43.75	121.00	672.00	672.00	672.00	2.49	3.00	2.21	2.49
	47.50	195.25	69.00	46.50	48.27	138.50	672.00	672.00	3.15	2.72	2.42	2.63
	40.25	93.75	40.75	53.00	53.00	140.75	672.00	672.00	2.44	2.72	3.07	2.62
	40.00	73.50	84.75	65.50	50.75	139.25	672.00	672.00	2.73	2.79	1.62	2.72
	48.50	160.00	190.75	42.25	42.25	184.75	672.00	672.00	2.74	2.79	3.16	5.50
	38.75	81.25	77.75	58.00	52.75	138.25	672.00	672.00	3.06	2.90	2.45	3.94
	44.50	118.75	115.50	90.00	47.75	104.50	672.00	672.00	2.19	2.32	2.20	1.87
	32.75	86.00	158.75	71.25	50.25	672.00	672.00	672.00	2.37	2.75	2.93	3.39
	41.00	78.00	72.50	53.00	54.00	130.25	672.00	672.00	2.70	2.75	2.83	2.95
	30.75	109.00	68.75	69.25	57.50	136.25	672.00	672.00	2.68	3.30	3.33	2.92
	39.25	82.75	77.50	62.25	49.00	129.00	672.00	672.00	2.60	2.46	2.89	2.77
	38.25	63.25	64.50	44.00	65.50	208.00	672.00	672.00	2.67	2.91	2.82	3.21
	36.50	155.50		40.75	60.75	138.50	672.00	672.00	2.60	2.68		2.67
	43.50	62.75		53.00	69.00	124.00	672.00	672.00	3.22	3.35		3.26
	32.75	108.75		44.50	47.25	146.25	672.00	672.00	2.40	2.69		1.96
	38.25	46.75		41.25	75.75	200.00	672.00	672.00	2.75	2.60		3.50
	35.50	103.75		70.00	49.25	135.75	672.00	672.00	2.59	2.66		2.63
	41.75	81.00		42.00	44.00	165.50		672.00	2.53	2.73		2.44
	38.25	59.25		131.75	66.00	120.75		672.00	2.68	2.67		2.20
Post-levofloxacin	−	+	+	−	−	+	+	−	−	+	+	−
Post-AVA	−	−	+	−	−	−	+	−	−	−	+	−
Pre-AVA	−	−	−	+	−	−	−	+	−	−	−	+

	Fig 10D				Fig 10E				Fig 10F			
	Mean SIBT (degrees above baseline)				Number of SIBTs				SIBT duration			
	1.89	1.73	1.54	2.27	1	5	1	1	4.25	76.75	5.00	34.50
	1.91	1.92	1.57	1.54	1	3	1	5	26.25	28.75	8.25	50.40
	1.95	2.08	1.68	1.65	3	1	2	3	37.50	17.25	29.00	54.75
	2.00	2.04	1.96	1.78	1	1	3	3	5.25	27.00	27.75	35.00
	2.15	1.60	1.24	1.72	1	2	1	2	8.25	45.25	7.50	33.00
	2.20	1.81	1.76	1.86	1	1	3	3	11.00	41.75	31.25	50.50
	2.28	2.21	1.99	2.59	1	2	2	3	12.75	30.50	10.00	32.75
	1.62	1.80	1.48	1.41	1	1	2	2	13.75	18.25	20.50	9.00
	2.01	1.98	1.76	2.42	1	2	3	4	15.50	44.00	15.25	34.00
	2.12	1.24	1.78	2.22	1	1	3	3	23.75	37.75	15.00	26.00
	2.20	2.41	2.48	1.98	1	3	1	2	9.75	49.25	19.00	19.50
	2.13	1.58	2.27	1.74	1	2	2	5	7.00	48.75	17.75	26.25
	1.93	1.94	2.14	2.00	1	2	2	10	13.25	51.00	11.00	99.00
	1.77	1.96		1.49	1	1		3	26.25	40.00		45.75
	3.01	2.58		2.09	1	1		4	2.75	23.75		39.25
	1.89	1.95		1.52	1	1		3	15.50	25.50		12.50
	2.12	1.54		2.55	1	1		2	10.50	31.00		19.25
	1.90	1.87		2.04	1	1		2	4.75	53.50		21.50
	1.76	1.82		1.50	1	1		3	8.25	44.00		45.00
	1.71	1.89		1.54	1	2		2	14.75	40.75		11.50
Post-levofloxacin	−	+	+	−	−	+	+	−	−	+	+	−
Post-AVA	−	−	+	−	−	−	+	−	−	−	+	−
Pre-AVA	−	−	−	+	−	−	−	+	−	−	−	+

Table 3. Summary of MCM efficacy and outcomes following Bcbva CA or CI aerosol exposure. MCM were administered as described in Materials and Methods. *Indicates animal succumbed immediately after treatment. Animal data was censored. #Indicates a telemetry transponder failed, and no temperature data was collected. βIndicates SIBT occurred following completion of treatment course. ^Indicates quantified values were not statistically different than pre-administration samples via pair-wise comparison. NA indicates not applicable. †Indicates seroconversion occurred before exposure. **All data are shown in the Table.**

Fig 11. Terminal bacterial burden in animals that succumbed following the cessation of levofloxacin treatment and non-treated controls. The CFU/mL in blood or broncho-alveolar lavage (BAL) samples and CFU/g of lung, liver, or spleen tissues of terminal untreated animals and animals that died after completion of levofloxacin treatment were quantified for Bcbva CA (A), or Bcbva CI (B). + Indicates treatment. – Indicates no treatment. Pair-wise comparisons were performed with a 2-way Student's T-test comparing bacterial titers between tissues harvested from levofloxacin-treated animals and control. * Indicates $P < 0.05$. ** Indicates $P < 0.01$. Only statistically significant comparisons are shown.

	Fig 11A	Bcbva CA									
		CFU/mL				CFU/g					
		9.00E+07	5.67E+02	1.08E+06	5.67E+02	2.47E+09	2.73E+06	1.73E+08	5.56E+04	1.10E+09	0.00E+00
		8.33E+06	3.07E+06	4.60E+03	5.47E+04	8.17E+08	3.29E+07	6.11E+07	5.46E+07	8.47E+07	1.52E+08
		4.93E+07	1.32E+06	1.17E+05	4.77E+03	1.33E+07	2.48E+07	6.14E+07	6.25E+05	6.30E+07	2.95E+06
		1.23E+07	2.27E+04	7.40E+04	0.00E+00	4.93E+08	3.67E+05	3.17E+08	7.94E+03	1.01E+08	4.17E+04
		6.63E+07	0.00E+00	2.13E+03	4.83E+04	3.83E+07	2.67E+08	5.00E+06	5.27E+07	1.20E+08	3.67E+07
		7.13E+07	6.00E+06	7.50E+05	7.30E+05	1.83E+09	4.78E+08	2.89E+08	2.69E+08	2.54E+08	1.55E+08
		8.20E+06	4.03E+06	5.30E+04	1.02E+05	4.53E+07	7.59E+07	1.09E+08	4.83E+07	1.23E+08	1.48E+08
		2.37E+07	8.00E+07	2.60E+03	2.57E+04	1.18E+07	3.28E+07	2.08E+07	6.19E+07	8.67E+07	7.33E+07
		9.17E+07	1.19E+04	1.87E+05	7.17E+04	2.77E+08	1.07E+07	6.83E+08	2.42E+06	1.77E+08	2.92E+06
		6.03E+06	2.33E+04	7.43E+04	1.09E+05	8.47E+08	9.08E+07	7.88E+07	1.40E+08	1.18E+08	1.63E+08
		8.47E+06	2.40E+05	2.47E+04	6.90E+04	2.28E+08	2.13E+07	1.10E+08	8.20E+06	3.28E+08	4.45E+06
		3.17E+06	1.13E+07	2.80E+03	1.00E+03	4.22E+07	4.67E+07	2.00E+07	4.62E+07	1.01E+08	8.00E+07
			1.10E+06		4.77E+03		3.86E+07		3.42E+07		3.89E+07
			0.00E+00		2.20E+05		1.23E+09		1.90E+08		1.98E+08
			7.77E+07		9.93E+03		8.44E+07		1.91E+07		4.83E+07
			8.70E+04		1.35E+04		2.08E+07		3.50E+06		1.57E+07
			4.67E+07		2.43E+04		5.57E+07		9.71E+07		2.63E+08
			2.40E+04		2.77E+04		4.48E+07		7.47E+07		7.40E+07
			0.00E+00		4.90E+04		1.25E+08		1.48E+08		8.40E+07
Post-levofloxacin		–	+	–	+	–	+	–	+	–	+
		Blood		BAL		Lung		Liver		Spleen	

	Fig 11B	Bcbva CI									
		CFU/mL				CFU/g					
		4.77E+06	3.33E+01	4.67E+04	2.03E+03	1.28E+08	2.49E+06	6.90E+07	0.00E+00	2.47E+08	0.00E+00
		3.70E+07	9.57E+05	4.67E+04	4.60E+04	6.77E+08	4.18E+07	1.43E+09	1.60E+07	1.32E+08	3.69E+07
		2.27E+08	6.57E+06	3.07E+05	5.23E+03	2.03E+09	2.78E+08	3.25E+08	1.42E+07	1.76E+08	1.03E+07
		7.10E+07	1.25E+07	2.00E+05	8.17E+04	1.67E+08	4.70E+07	1.05E+09	5.79E+07	2.70E+08	9.21E+07
		2.63E+06	2.57E+07	8.63E+03	3.33E+05	6.68E+08	2.74E+08	1.22E+08	4.16E+07	9.87E+07	8.88E+07
		7.77E+06	3.60E+04	5.27E+03	5.13E+03	3.49E+07	4.80E+06	4.06E+07	1.35E+05	5.52E+07	1.62E+05
		7.23E+06	1.34E+06	1.01E+04	1.30E+03	3.07E+07	4.39E+07	2.97E+07	9.64E+06	6.33E+07	6.27E+06
		1.22E+08	1.91E+07	2.37E+05	4.03E+04	4.67E+08	7.86E+07	2.47E+09	6.27E+07	6.44E+08	3.50E+07
		2.27E+07	7.07E+06	6.73E+04	2.60E+03	3.86E+08	6.95E+07	1.16E+08	3.63E+07	3.88E+08	6.67E+07
		2.70E+07	4.27E+06	5.83E+04	8.10E+03	7.30E+08	1.97E+07	2.35E+08	1.54E+07	1.36E+08	6.15E+07
		6.40E+07	3.93E+07	5.60E+05	4.70E+05	5.80E+08	1.81E+08	8.73E+08	1.07E+08	7.61E+07	1.62E+08
		6.97E+06	7.87E+04	6.00E+04	3.67E+02	7.75E+06	5.15E+07	8.43E+07	5.03E+07	4.38E+07	6.11E+07
			6.80E+05		7.33E+04		4.40E+07		4.17E+07		9.52E+07
			5.17E+07		5.10E+03		2.05E+07		7.00E+07		1.09E+08
			6.10E+07		8.00E+04		9.29E+07		1.08E+08		8.48E+07
			9.67E+05		1.13E+04		4.61E+07		6.76E+07		1.20E+08
			6.90E+06		5.47E+04		1.47E+08		1.48E+07		3.98E+07
			8.80E+06		2.23E+05		7.70E+07		1.79E+08		4.59E+07
Post-levofloxacin		-	+	-	+	-	+	-	+	-	+
		Blood		BAL		Lung		Liver		Spleen	

Fig 12. Quantification of virulence factors in MCM-treated animals following aerosol exposure to Bcbva CA and CI. Hyaluronic acid (A) and protective antigen (B) were quantified in serum samples upon death or from survivors at the end of the observation period. Symbols indicate individual data points. Horizontal line indicates the mean. For control and levofloxacin-only treated animals the data values are from non-survivor terminal samples. For dual treatment with PEP and Post-AVA, and pre-AVA animals, the data values represent quantification of day 28 samples since no animals succumbed during the observation period. Collectively, comparisons were made between survivors and non-survivors as a function of treatment regimen. + Indicates treatment. – Indicates no treatment. One-way ANOVA was performed followed by a Tukey's Test to perform pair-wise comparisons between the MCM treatment groups and control. * Indicates P < 0.05. ** Indicates P < 0.01. *** Indicates P < 0.001. **** Indicates P < 0.0001. Only statistically significant comparisons are shown.

	Fig 12A	hyaluronic acid (ng/mL)							
		5.71E+05	1.93E+03	2.63E+00	3.76E-01	1.30E+06	1.10E+05	9.05E+01	4.81E+00
		1.06E+06	6.93E+04	1.22E+01	9.23E+01	1.48E+05	2.56E+05	2.40E+03	5.26E+01
		9.86E+05	2.96E+05	1.46E+01	1.00E+03	5.38E+05	4.44E+05	2.21E+01	2.75E+00
		1.37E+06	1.25E+05	5.84E+01	1.20E+01	4.94E+05	2.39E+05	1.47E+02	1.17E+01
Post-levofloxacin		–	+	+	–	–	+	+	–
Post-AVA		–	–	+	–	–	–	+	–
Pre-AVA		–	–	–	+	–	–	–	+
		Bcbva CA				Bcbva CI			
	Fig 12B	protective antigen (ng/mL)							
		9.02E+03	3.88E+03	3.17E+01	4.59E+01	4.73E+03	8.32E+03	7.71E+01	1.21E+02
		1.33E+04	1.42E+03	2.99E+01	5.71E+01	1.71E+04	1.02E+04	5.72E+01	1.08E+02
		1.27E+04	4.40E+03	3.11E+01	*-3.905882**	4.45E+03	7.07E+03	9.69E+01	1.12E+02
		1.09E+04	7.46E+03	7.38E+01	4.69E+01	4.73E+03	5.12E+03	7.35E+01	1.28E+02
Post-levofloxacin		–	+	+	–	–	+	+	–
Post-AVA		–	–	+	–	–	–	+	–
Pre-AVA		–	–	–	+	–	–	–	+
		Bcbva CA				Bcbva CI			

Fig 13. Seroconversion against protective antigen. Antibodies that bind to protective antigen were measured in serum samples from rabbits exposed to Bcbva and treated with medical countermeasures (**A**). Seroconversion in MCM-treated animals. Only the animals that seroconverted are shown. Results for Bcbva CA and CI experiments were combined since a statistical difference was not observed as a function of challenge strain by ROUT outlier analysis (post-levofloxacin survivors) and pair-wise comparisons with a two-way Student's T-test (post-levofloxacin and AVA; Pre-AVA). The survival outcome for each treatment group is indicated. + Indicates treatment occurred. – Indicates treatment did not occur. Symbols indicate individual data points. Horizontal line indicates the mean. One-way ANOVA was performed followed by a Tukey's Test to perform pair-wise comparisons between the MCM treatment groups, except for the post-levofloxacin non-survivor group (too few data point for statistical analysis). Only statistically significant comparisons are shown. Blood serum samples from $INHLD_{50}$ experiments were quantified for antibody that binds protective antigen (**B**). Pair-wise comparisons were performed with a 2-way Student's T-test comparing survivors and non-survivors. NS indicates non-survivors. S indicates survivors.

	Fig 13A	protective antigen (ng/mL)							
		Survivors			Non-survivors				
		5.00E+06	4.04E+06	6.86E+06	1.02E+04				
		7.97E+06	1.49E+07	5.93E+06	7.93E+03				
		1.55E+06	1.51E+06	2.48E+06					
			1.60E+06	3.34E+06					
			1.24E+06	3.29E+06					
			8.68E+06	3.49E+06					
			5.98E+06	6.25E+06					
			4.38E+06	7.83E+06					
				1.23E+06					
				1.17E+06					
				1.58E+06					
				9.63E+05					
				3.86E+05					
				6.03E+05					
				1.50E+06					
				3.30E+06					
Post-levofloxacin		+	+	−	+				
Post-AVA		−	+	−	−				
Pre-AVA		−	−	+	−				
	Fig 13B	protective antigen (IgG U/mL)							
		28.93	173.96	472.33	204.47	172.89	423.01	260.24	173.92
		343.40	246.92	613.84	548.18	356.63	207.35	290.36	157.65
		337.11	476.79	852.83	188.43	1094.58	374.82	61.45	78.73
		623.27	209.18	1815.09	425.22	-4.82	832.95	1615.66	609.16
		Non-survivor		Survivor		Non-survivor		Survivor	
		Bcbva CA				Bcbva CI			

Supplemental Fig 1. Mean time to SIBT or death following aerosol exposure to Bcbva CA (left) and CI (right). The time to SIBT (A, B) and death (C, D) are indicated with a symbol. One-way ANOVA was performed followed by a Tukey's multiple comparison test to perform pair-wise comparisons between each does group. Animals that survived the entire observation period were represented by data points at 500 hours post-exposure for comparative purposes. * indicates a P-value of < 0.05. ** indicates a P-value of < 0.01. *** indicates a P-value of < 0.001. **** indicates a P-value of < 0.0001. Two groups of 8 animals were challenged with 1.8 X 10^5 CFU Bcbva CI. These groups were combined for data analysis (16 animals in this group). Dose in CFU.

Supplementary Fig 1A					
	Time to SIBT Bcbva CA (h)				
	504.00	354.75	504.00	504.00	61.75
	504.00	59.75	504.00	504.00	58.00
	44.25	37.50	504.00	250.00	83.00
	504.00	49.00	504.00	504.00	49.50
	241.50	34.50	504.00	504.00	50.75
	56.00	236.25	504.00	504.00	42.75
	288.25	149.50	417.50	504.00	64.00
	45.00		504.00	504.00	53.50
Dose	3.2 x 10^5	4.8 x 10^6	2.7 x 10^4	7.9 x 10^4	3.9 x 10^7

Supplementary Fig 1B				
	Time to SIBT Bcbva CI (h)			
	68.25	504	63.5	38.25
	86.5	504	51.25	47.5
	103	504	164	40
	92.25	504	504	38.75
	504	504	45.25	28.75
	504	504	42.75	40.25
	42	504	37	48.5
	64.5	504		44.5
		504		
		504		
		504		
		57.25		
		149.25		
		504		
		224.25		
Dose	6.3 x 10^5	1.8 x 10^5	1.9 x 10^6	4.9 x 10^7

Supplementary Fig 1C					
	Time to death Bcbva CA (h)				
	504.00	374.50	504.00	504.00	61.75
	504.00	66.25	504.00	504.00	58.00
	72.25	62.25	504.00	295.25	83.00
	504.00	86.25	504.00	504.00	49.50
	300.75	64.50	504.00	504.00	50.75
	106.00	248.50	504.00	504.00	42.75
	294.00	181.00	436.75	504.00	64.00
	84.25		504.00	504.00	53.50
Dose	3.2 x 10^5	4.8 x 10^6	2.7 x 10^4	7.9 x 10^4	3.9 x 10^7

Supplementary Fig 1D				
	Time to death Bcbva CI (h)			
	71.25	504.00	70.50	43.50
	105.75	504.00	61.50	121.00
	160.75	504.00	200.25	48.27
	106.75	504.00	504.00	53.00
	504.00	504.00	49.75	57.50
	504.00	504.00	51.25	49.00
	56.25	504.00	82.50	65.50
	76.00	504.00		60.75
		504.00		
		504.00		
		504.00		
		69.75		
		153.00		
		504.00		
		252.00		
Dose	6.3 x 10^5	1.8 x 10^5	1.9 x 10^6	4.9 x 10^7

Supplemental Fig 2. Time between onset of SIBT and death/humane euthanasia. The individual data points with the mean are plotted for the time between SIBT and death for rabbits exposed to Bcbva isolates CA (**A**) or CI (**B**). One-way ANOVA was performed followed by a Tukey's multiple comparison test to perform pair-wise comparisons between each dosage group. No significant differences were found between groups. Dosage groups 2.7×10^4 and 7.9×10^4 with CA could not be compared due to n = 1. Dose in CFU.

Supplemental Fig 2A							Supplemental Fig 2B				
	Time SIBT to death Bcbva CA (h)						Time SIBT to death Bcbva CI (h)				
	28.00	19.75	19.25	45.25	26.25			3	12.5	7	5.25
	59.25	6.50			11.75			19.25	3.75	10.25	73.5
	50.00	24.75			42.75			57.75	27.75	36.25	8.27
	5.75	37.25			6.25			14.5		4.5	14.25
	39.25	30.00			12.25			14.25		8.5	28.75
		12.25			10.25			11.5		45.5	8.75
		31.50			11.25						17
					19.75						16.25
Dose	3.2×10^5CFU	4.8×10^6CFU	2.7×10^4CFU	7.9×10^4CFU	3.9×10^7CFU		Dose	6.3×10^5	1.8×10^5	1.9×10^6	4.9×10^7

Supplemental Fig 3. Colony morphologies of Bcbva. Representative colonies of each colony morphology are shown from pre-aerosol, post-aerosol, and in vivo samples for Bcbva CA and Cl. Heat shocked spores prior to (panel 1) and following (panel 2) aerosolization with a 3-Jet Collison nebulizer. The colonies are average in size, mucoid, and display a fried egg appearance. Pre-terminal bacteremic blood samples (panel 3 and 4). The colonies appear large, matte, round or amorphous, and indicative of swarming motility. The colony in the top right panel (4) was sampled with a loop to demonstrate the tackiness of the colony. Terminal bacteremic blood samples (panels 5-8), which also represent the colony morphologies obtained from lung, liver, and spleen homogenates (have similar appearance). The colonies are average in size and either highly mucoid with fried egg-like appearance (predominant morphology) or matte and round (infrequent). Terminal bacteremic blood samples. The colonies are either average in size and round with matte or highly mucoid presentations or small, round, and mucoid. Images represent colony morphologies observed for both isolates. The images were scaled so that the vertical diameters of agar plates are identical among all images. **All data are shown in the Figure**.
