# Supplementary figures and images for: Bacillus cereus biovar anthracis causes inhalational anthrax-like disease in rabbits that is treatable with medical countermeasures
Source: PLoS Negl Trop Dis. 2025 Apr 7;19(4):e0012973. doi: 10.1371/journal.pntd.0012973 (PMC12005533; doi:10.1371/journal.pntd.0012973)

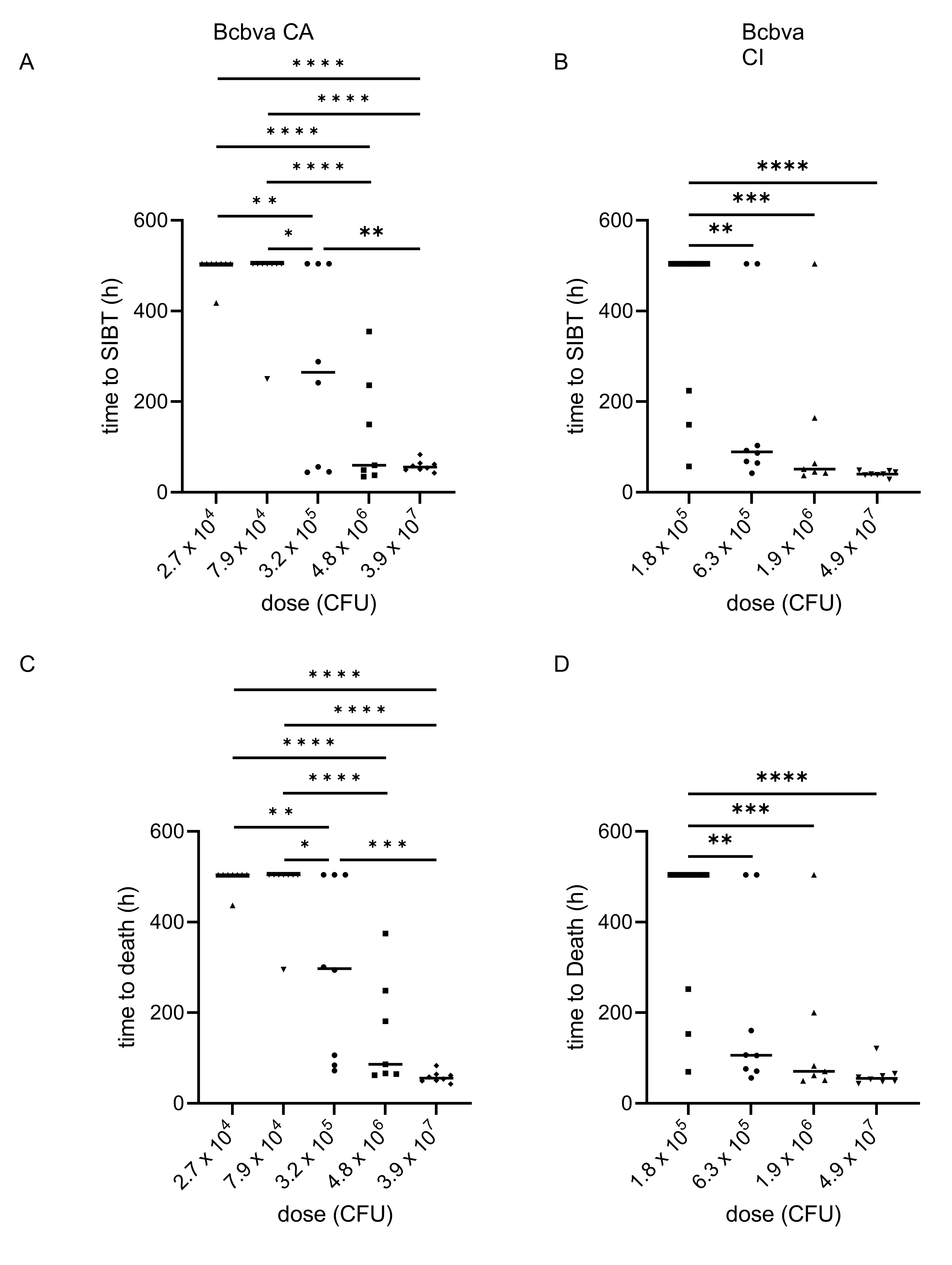

Supplement: S1 Fig — The time to SIBT (A, B) and death (C, D) are indicated with a symbol. One-way ANOVA was performed followed by a Tukey’s multiple comparison test to perform pair-wise comparisons between each does group. Animals that survived the entire observation period were represented by data points at 500 hours post-exposure for comparative purposes. * indicates a P-value of < 0.05. ** indicates a P-value of < 0.01. *** indicates a P-value of < 0.001. **** indicates a P-value of < 0.0001. Two groups of 8 animals were challenged with 1.8 X 105 CFU Bcbva CI. These groups were combined for data analysis (16 animals in this group). (TIF) [file pntd.0012973.s002.tif]

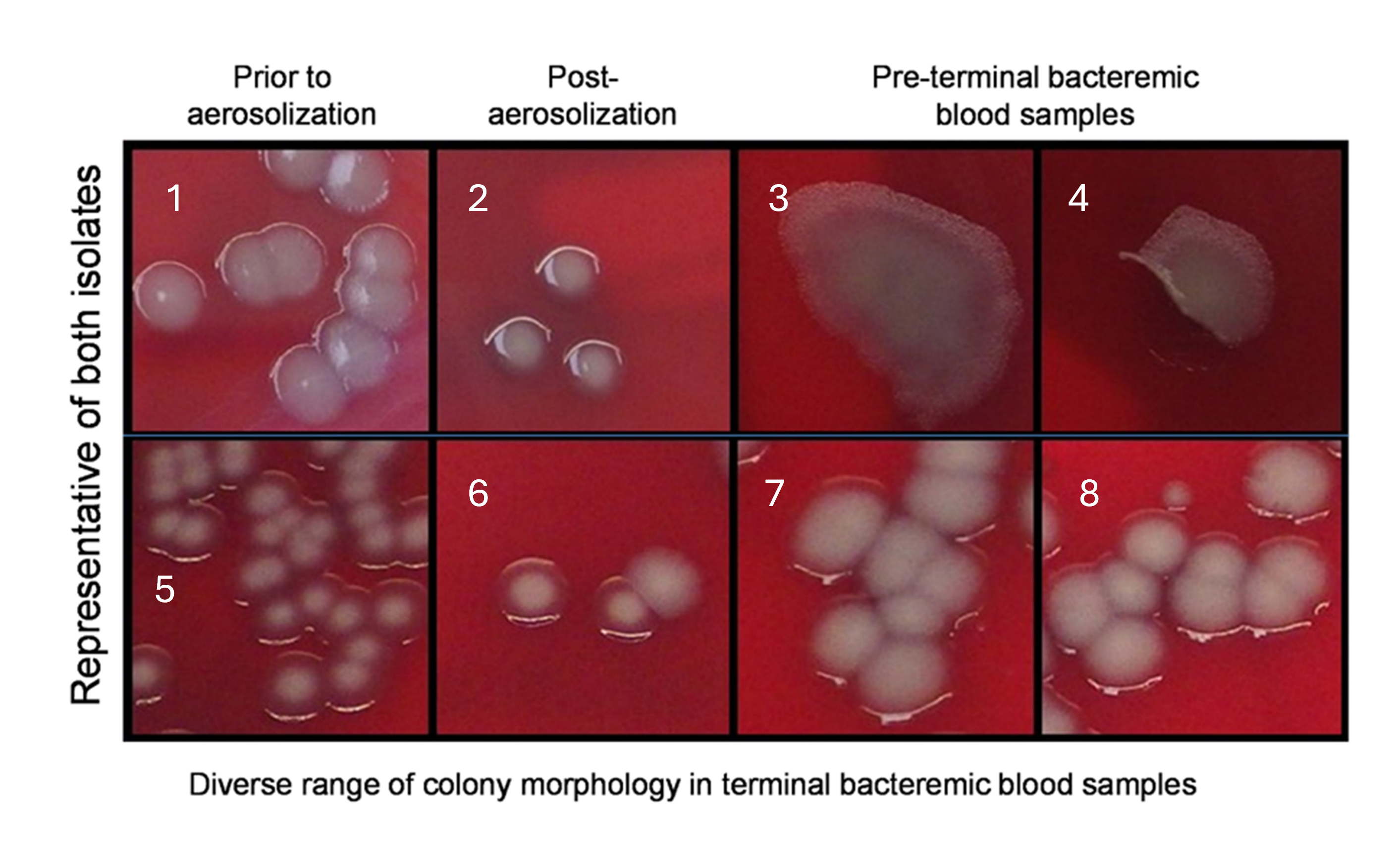

Supplement: S3 Fig — Representative colonies of each colony morphology are shown from pre-aerosol, post-aerosol, and in vivo samples for Bcbva CA and Cl. Heat shocked spores prior to (panel 1) and following (panel 2) aerosolization with a 3-Jet Collison nebulizer. The colonies are average in size, mucoid, and display a fried egg appearance. Pre-terminal bacteremic blood samples (panel 3 and 4). The colonies appear large, matte, round or amorphous, and indicative of swarming motility. The colony in the top right panel (4) was sampled with a loop to demonstrate the tackiness of the colony. Terminal bacteremic blood samples (panels 5–8), which also represent the colony morphologies obtained from lung, liver, and spleen homogenates (have similar appearance). The colonies are average in size and either highly mucoid with fried egg-like appearance (predominant morphology) or matte and round (infrequent). Terminal bacteremic blood samples. The colonies are either average in size and round with matte or highly mucoid presentations or small, round, and mucoid. Images represent colony morphologies observed for both isolates. The images were scaled so that the vertical diameters of agar plates are identical among all images. (TIF) [file pntd.0012973.s004.tif]
