# Supplementary material for: Bacillus cereus biovar anthracis causes inhalational anthrax-like disease in rabbits that is treatable with medical countermeasures
Source: PLoS Negl Trop Dis. 2025 Apr 7;19(4):e0012973. doi: 10.1371/journal.pntd.0012973 (PMC12005533; doi:10.1371/journal.pntd.0012973)
Supplement: Attachment — Submitted filename: review.pdf [file pntd.0012973.s005.pdf]

02/12/25 Review for PNTD-D-25-00035
Bacillus cereus biovar anthracis causes inhalational anthrax-like disease in rabbits that is treatable with medical countermeasures

This is a well written paper full of data describing infections and AVA vaccine protection against two emerging anthrax-causing Bcbva strains.

Line 82- Jiranantasak et al 2024 https://journals.plos.org/plosntds/article?id=10.1371/journal.pntd.0012779# recently tested Sterne Vaccine against older strains of Bcbva in the context of other work and this one should be mentioned,.
Line 122- I am unfamiliar with the gene names *saspB*. Is this *sspB* gene target for the small acid soluble protein? provide a reference.
Line 203-Hyaluronic acid released by the host as animals succumb to Bcbva is not specific for the Bcbva capsule is there data supporting the source of the HA as Bcbva?
Line 324-MTD is irrelevant to dose? The only doses summarized are a 10-fold difference. I'm not sure that is a wide enough range to claim MTD of B. anthracis spores are not dependent on dose. Time to death does decrease as doses increase. There are several papers out there that show that. Here are 3
https://pmc.ncbi.nlm.nih.gov/articles/PMC7350313/ ,
https://www.sciencedirect.com/science/article/abs/pii/0264410X94900272?via%3Dihub,
https://journals.plos.org/plosntds/article?id=10.1371/journal.pntd.0012779#

Figure 1 seems unnecessary, suggest move to supplementary
Figure 2- what are the MLD50s and confidence intervals of CA and CI as determined by ProBit, list them in the manuscript text.
Figure 4- There is essentially no difference between SIBT and TTD. Is a graph for SIBT necessary? This fact is well known as anthrax infected animals succumb rapidly sometimes with no symptoms.
Figure 5- This figure is confusing, -2 in comparison to TTD? Is there a more logical way to show this? I understand that the goal is to show that regardless of dose bacteremia prior to death occurs under same dynamics. Maybe a multi-part figure with graph for each dose and day 0 as day of challenge?

Figure 6 and 7 can be combined, as they describe same results from the two strains with no substantial differences in methods or interpretation

Figure 8 in vivo data for Ba would be great to have here, especially for the hyaluronic acid. This would show whether the hyaluronic acid detected is specific to release from Bcbva and not host.

Figure 9- in A and B and Figure 10 A and B- days might make more sense to the reader since the minimum is around 2 days and max is nearly 10, this might also impact statistical analysis but extending survival by days would undoubtedly be the more successful measure.

Figure 9 and 10- It is also really confusing to show that AVA post and pre-treated animals have an SIBT and TTD even though most survived the study. I understand that you are trying to compare statistically. Is there another way to show survivors vs non-survivors in this figure. survivors as white circles so that these factors can be visually attributed to survival?

Line 494- data not shown…why? There are 13 figures and several supplementary, including the data in supplementary is recommended.

Line 591-593 The studies show that PEP with levofloxacin is not able to protect rabbits. The comments on these lines needs to be edited from '*PEP regimens with levofloxacin, with or without AVA, are protective against aerosol exposure to lethal doses of either Bcbva CA or CI'*

Line 595: Some discussion of Sterne efficacy would be appropriate here. Sterne vaccination was effective in Jiranantasak et al though earlier strains of Bcbva were more virulent than those tested here.
