# Supplementary material for: Bacillus cereus biovar anthracis causes inhalational anthrax-like disease in rabbits that is treatable with medical countermeasures
Source: PLoS Negl Trop Dis. 2025 Apr 7;19(4):e0012973. doi: 10.1371/journal.pntd.0012973 (PMC12005533; doi:10.1371/journal.pntd.0012973)
Supplement: Attachment — Submitted filename: Response to reviewers.docx [file pntd.0012973.s007.docx]

Dear PLOS NTD,

Thank you for reviewing our manuscript. Below are the reviewer comments and our responses to them are in red text.

Angelo Scorpio, corresponding author

7 March 2025

**Reviewers' comments:**

Reviewer's Responses to Questions

**Key Review Criteria Required for Acceptance?**

As you describe the new analyses required for acceptance, please consider the following:

**Methods**:

-Are the objectives of the study clearly articulated with a clear testable hypothesis stated?

-Is the study design appropriate to address the stated objectives?

-Is the population clearly described and appropriate for the hypothesis being tested?

-Is the sample size sufficient to ensure adequate power to address the hypothesis being tested?

-Were correct statistical analysis used to support conclusions?

-Are there concerns about ethical or regulatory requirements being met?

Reviewer #1: The study objective and the design were clear enough but quite similar to the work of Ionin et al 2013. The statistical analysis used is correct, the facility where the work was done was accredited and the experiments performed according to standard guidelines. However, the following required response from the authors;
1. The cultivation of the study isolate, B. cereus biovar anthracis as described in line 104 should require biosafety protocols given the virulence status of the bacterium. What were the precautions (IPC) put in place?

Response: The statement on growth conditions was revised to indicate that *B. cereus* Biovar *anthracis* strains were grown in a Biosafety Level 3 (BSL-3) laboratory

2. How were the euthanized rabbits disposed of (line 152)?

Response: All animals were disposed of per standard ABSL-3 practices at the conclusion of the study. A sentence was added to indicate this.

3. There is need to sufficiently explain how the incoming air into the 3-jet collison chamber was adequate enough such that low/poor Oxygen threshold would not have contributed to the death of the animals.

Response: A sentence was added to indicate oxygen threshold was adequate during the exposure: “During the exposure, animals were monitored by veterinary technical staff for normal breathing.” In the Animal inhalational challenge system section a sentence was added: “A continuous flow of HEPA-filtered house air was supplied to the nebulizer at 7.5 liters per minute to allow animals to breath normally.”

4. What anthrax-like symptoms were presented by the animals?

Response: Line 293 in the original submission indicates the following signs of disease: “ruffled fur, lethargy, loss of appetite, reduced bowel activity, and accumulation of dried nasal mucus.”

5. Was the ELISA direct or indirect?

Response: Multiple ELISA assays were used for the study. The PA ELISA was a direct assay kit – the description was revised to indicate this.

Reviewer #2: In general, the methods are well described, with one exception (see comments).

Reviewer #3: Mostly, see specific comments

**Results**:

-Does the analysis presented match the analysis plan?

-Are the results clearly and completely presented?

-Are the figures (Tables, Images) of sufficient quality for clarity?

Reviewer #1: The authors presented the results in tables and images that are of sufficient quality although clearer explanations are needed on the operation of spore delivery instruments.

Reviewer #2: Overall, the results are clearly and completely presented. However, there are some issues which need clarification or can be improved (see comments).

Reviewer #3: Mostly, some figures are overly complex and could use some clarification, others some consolidation.

**Conclusions**:

-Are the conclusions supported by the data presented?

-Are the limitations of analysis clearly described?

-Do the authors discuss how these data can be helpful to advance our understanding of the topic under study?

-Is public health relevance addressed?

Reviewer #1: The authors did not draw any specific conclusions to underline the study findings. The conclusion should be presented as a separate sub-heading. The limitations of analysis should also be distinctly described.

Response: We believe the first paragraph of the Discussion section draws conclusions and main findings of the study.

Reviewer #2: The conclusions are supported by the data presented. The main outcome of the study is the fact that there are usually no differences between inhalational infections with Bcbva and B. anthracis, also concerning the efficacy of MCM. However, two differences were observed which should be discussed in more detail (see comments).

Reviewer #3: Yes, a little more regarding previous animal models and other modes of vaccinations should be dicussed.

Response: We cited previous work on Bcbva and *B. anthracis* animal models of infection. We focused on AVA vaccination since it is PA-based and is used to vaccinate individuals who are at risk of exposure to *B. anthracis*. We cited previous vaccination studies that utilized PA and formalin inactivated *B. anthracis* spores.

**Editorial and Data Presentation Modifications?**

Use this section for editorial suggestions as well as relatively minor modifications of existing data that would enhance clarity. If the only modifications needed are minor and/or editorial, you may wish to recommend “Minor Revision” or “Accept”.

Reviewer #1: Minor revision

Reviewer #2: Methods:
The growth conditions to assess the in vitro expression of virulence factors (HA, PA, PDGA) should be added: medium, incubation time, with or without CO2?

Response: Thank you for noting this. Text was updated in Methods to specify growth conditions.

Results:
Fig. 2 A and B: The curves are wrong. The highest infection doses result in highest probability of survival, this cannot be true.

Response: Thank you for noting this. The Y-axis label was revised in Figure 2B and 2C to indicate ‘probability of lethality’.

Lines 285 and 287: The authors probably refer to Fig. 3 and Fig. S2, not to Fig. 4 and Fig. S1.

Response: Thank you for noting this. These were changed to Fig 3. The supplemental figures cited were not relevant to these points and so were removed.

Fig. 3: The exposure doses should be indicated in the figure or in the legend. The figure would be easier to understand if the hours would be counted from the timepoint of exposure (like SIBT and ToD) and not from the start of temperature registration.

Response: Thank you for the suggestion. Doses were added to each representative temperature response. The telemetry graphs were meant as a representative illustration of temperatures during baseline (prior to challenge) and following challenge. We include baseline temperatures to give an idea of fluctuations and to establish 3 SD from baseline. The SIBT is subtracted from the time of challenge. This representation is also consistent with our previous publication (J Am Assoc Lab Anim Sci. 2017;56(6):792-801.)

Fig. 4 D: 5/8 animals (Fig. S1) infected with Bcbva CI survived. This would be 62.5% and not 80% as indicated in the figure.

Response: Thank you for noting this. Two groups of 8 animals were challenged with 1.8 X 10^5^ CFU Bcbva CI. These groups were combined for data analysis (16 animals in this group). Three animals succumbed, resulting in 13/16 survival (81%). The Fig 4 and S1 legends were revised to indicate this.

Table 2: What are the numbers in brackets?

Response: Thank you for noting this. These are 95% confidence intervals. The Table 2 legend was updated to indicate this.

Line 350: it would be better to write “colonies from cultured spores” instead of “cultured spores”.

Response: Thank you. Text was updated accordingly.

Line 352: did all colonies (100%) derived from animals 1-2 days prior to death display the large matte phenotype or was this only observed in a fraction of colonies?

Response: Yes, all of the colonies derived from blood 1-2 days prior to death displayed this morphology. Text was updated to clarify.

Fig. S3: The caption is difficult to understand. It would be easier if the photos were numbered and the explanation in the caption would refer to the numbers.

Response: Supplemental Figure 3 was revised with numbers added to each panel. The caption now references the panel number.

Fig. 8: The in vitro growth conditions must be described (see Methods). Due to a mutation in the hasA gene, B. anthracis should not be able to express a HA capsule. Did the authors perform the ELISA with a negative control (medium alone) which also shows this background expression? HA is a component of the extracellular matrix of animal tissues. The high in vivo HA concentration in serum might be the result of the animal and not the bacterial HA. An appropriate control (HA ELISA with serum of non-infected animals) must be performed.

Response: Yes, we compared a negative control sample with the *B. anthracis* samples and they were not statistically different. We tested pre-challenge serum for each animal and were not able to detect HA in any of the samples. The Figure 8 caption was updated to reflect this.

Fig. 12: Only 4 data points per group are shown, but the experiment included 20 animals per group (as shown in Fig. 9 and 10). Did the authors analyze HA and PA expression of only 4 animals per group?

Response: Yes, we analyzed 4 samples per group which was sufficient to allow statistical comparisons. Symbols in Fig 12 indicate individual data points.

Conclusions:
Lines 562 ff: How do the authors explain the dose-dependent time to SIBT or death in Bcbva in contrast to B. anthracis? There seems to be one report for B. anthracis (ref. 35), but it is not consistently observed.

Response: We do not have an explanation for dose-dependent time to SIBT. We added text indicating that the observed motility of Bcbva may contribute to this observation.

Lines 567 ff: Bcbva colonies derived from animals 1-2 days prior to death displayed a large matte phenotype indicative of swarming motility. In contrast to Bcbva, B. anthracis is non-motile due to mutations in the flagellar gene cluster and would be unable to display this phenotype, regardless of differential gene expression. This should be discussed. It would be interesting to assess the colony morphology of a non-motile deletion mutant of Bcbva.

Response: Added text regarding motility of Bcbva and lack of motility in *B. anthracis*. We agree, it would be interesting to test a non-motile Bcbva variant for the large matte colony appearance.

Line 587: Why do the authors think that there is “some indication that the presence of PDGA capsule may not be essential during ... infection in NZW rabbits”? This was shown in mice with Bcbva lacking the PDGA capsule. Did the authors perform infections with Bcbva lacking the pXO2 capsule plasmid or is this just a conclusion drawn from the fact that a high concentration of HA was detected in rabbit serum?

Response: Thank you for noting this – the statement was revised for clarity. We did not perform infections with Bcbva lacking the pXO2 plasmids.

Acknowledgements, line 606: please indicate “Robert Koch Institute, Berlin, Germany”.

Response: Revised as indicated

Reviewer #3: See specific comments

**Summary and General Comments**:

Use this section to provide overall comments, discuss strengths/weaknesses of the study, novelty, significance, general execution and scholarship. You may also include additional comments for the author, including concerns about dual publication, research ethics, or publication ethics. If requesting major revision, please articulate the new experiments that are needed.

Reviewer #1: (No Response)

Reviewer #2: The study adds to the knowledge of Bcbva pathogenesis in an animal model (NZW rabbits) from which data were not yet available. MCM intervention which is used for treatment of inhalational anthrax caused by B. anthracis seems to be effective also against Bcbva.

Reviewer #3: This is a well written paper full of data describing infections and AVA vaccine protection against two emerging anthrax-causing Bcbva strains.

PLOS authors have the option to publish the peer review history of their article ([what does this mean?](https://urldefense.us/v3/__https:/track.editorialmanager.com/CL0/https:*2F*2Fjournals.plos.org*2Fplosntds*2Fs*2Feditorial-and-peer-review-process*23loc-peer-review-history/1/010f019503ccb4cc-c6a124c6-a01e-4ee5-a0d9-5578fc4bcfea-000000/F7BYuk5px2vCNuLscuTKb25iJDx-tq77AaxyDhIbfOo=198__;JSUlJSUl!!BClRuOV5cvtbuNI!H6wwyHTO6nC760Ok3CALhj_Z_iSMeSTFIOlUMzNcSFETj2uhP234a6iVelBQHmEt_Z_NWyowa2oDhrcWdO16WxI$)). If published, this will include your full peer review and any attached files.

If you choose “no”, your identity will remain anonymous but your review may still be made public.

**Do you want your identity to be public for this peer review?** For information about this choice, including consent withdrawal, please see our [Privacy Policy](https://urldefense.us/v3/__https:/track.editorialmanager.com/CL0/https:*2F*2Fwww.plos.org*2Fprivacy-policy/1/010f019503ccb4cc-c6a124c6-a01e-4ee5-a0d9-5578fc4bcfea-000000/QpvrUdMIwnUd3nrOuAwd4l5pVUgu4LAgD6Trc7DuQ40=198__;JSUl!!BClRuOV5cvtbuNI!H6wwyHTO6nC760Ok3CALhj_Z_iSMeSTFIOlUMzNcSFETj2uhP234a6iVelBQHmEt_Z_NWyowa2oDhrcW-Z1xUmo$).

Reviewer #1: **Yes:** Bamidele Tajudeen Akanji

Reviewer #2: No

Reviewer #3: No

[NOTE: If reviewer comments were submitted as an attachment file, they will be attached to this email and accessible via the submission site. Please log into your account, locate the manuscript record, and check for the action link "View Attachments". If this link does not appear, there are no attachment files.]

**Figure resubmission:**

While revising your submission, please upload your figure files to the Preflight Analysis and Conversion Engine (PACE) digital diagnostic tool, [https://pacev2.apexcovantage.com/](https://urldefense.us/v3/__https:/pacev2.apexcovantage.com/__;!!BClRuOV5cvtbuNI!H6wwyHTO6nC760Ok3CALhj_Z_iSMeSTFIOlUMzNcSFETj2uhP234a6iVelBQHmEt_Z_NWyowa2oDhrcWCwaYJN0$). PACE helps ensure that figures meet PLOS requirements. To use PACE, you must first register as a user. Registration is free. Then, login and navigate to the UPLOAD tab, where you will find detailed instructions on how to use the tool. If you encounter any issues or have any questions when using PACE, please email PLOS at [figures@plos.org](mailto:figures@plos.org). Please note that Supporting Information files do not need this step. If there are other versions of figure files still present in your submission file inventory at resubmission, please replace them with the PACE-processed versions.

**Reproducibility:**

To enhance the reproducibility of your results, we recommend that authors of applicable studies deposit laboratory protocols in protocols.io, where a protocol can be assigned its own identifier (DOI) such that it can be cited independently in the future. Additionally, PLOS ONE offers an option to publish peer-reviewed clinical study protocols. Read more information on sharing protocols at [https://plos.org/protocols?utm_medium=editorial-email&utm_source=authorletters&utm_campaign=protocols](https://urldefense.us/v3/__https:/plos.org/protocols?utm_medium=editorial-email&utm_source=authorletters&utm_campaign=protocols__;!!BClRuOV5cvtbuNI!H6wwyHTO6nC760Ok3CALhj_Z_iSMeSTFIOlUMzNcSFETj2uhP234a6iVelBQHmEt_Z_NWyowa2oDhrcWsVamGGE$)
